# Community first responders: A missing key to reducing the impact of injury and illness in low- and middle-income countries in the Western Pacific?

**DOI:** 10.5365/wpsar.2020.11.1.003

**Published:** 2021-06-22

**Authors:** Andrew Hodgetts, Peter Massey, Michelle Redman-MacLaren, Roxanne Bainbridge

**Affiliations:** a Central Queensland University, Cairns, Australia.; b Hunter New England Health, New Lambton, Australia.; c James Cook University, Cairns, Australia.

The higher burdens of morbidity and mortality in low- and middle-income countries (LMICs) in the Western Pacific Region (WPR) could be reduced if there were community first responders qualified in first aid and trained according to the local context. In the WPR, the leading causes of death of people aged 5–49 years are violence and injury, which claim the lives of  1 million people each year. (*1*) Emerging data highlight the burden of violence and injury in the Region, (*1*) but there are no reliable data to indicate the potential benefits of having community first responders. Community first responders might make a significant difference in the rates of mortality and morbidity associated with injury and with other health issues for which timely, effective first aid could help.

In LMICs in the WPR, the recognition and initiation of basic first-aid measures fall to the community because of limited access to formal health services. (*2*) In this Region, cardiovascular disease, complications of diabetes and respiratory diseases account for the majority of adult deaths, contribute to an increasing burden on the health systems and slow development. (*3*) Community first responders who are trained to identify these medical conditions could start targeted primary management and provide early reports to formal health service providers. Potentially, first responders could significantly reduce the harm of delayed treatment of diseases and injuries in their communities.

Community first responders have been reported to make a difference in LMICs in Iraq, Nigeria and South Africa. In South Africa, participants from a township in the Cape Town region who were given a 1-day training session provide effective basic first aid to members of their communities until professional ambulance services arrive. (*4*) In a similar programme in Ibadan, south-western Nigeria, drivers of commercial passenger vehicles were trained for 2 days in basic first aid techniques. (*5*) As commercial drivers are most likely to encounter motor vehicle accidents, they are able to provide immediate initial first aid. Both programmes established that laypeople can successfully complete first aid training programmes, demonstrate skills and retain knowledge, as shown in re-testing. (*4*, *5*) In Iraq, village first responders worked in partnership with trained paramedics to treat victims of motor vehicle accidents, and a significant reduction in mortality was recorded among people who received such pre-hospital care. (*6*)

In most LMICs, the burden of injury and illness affects not only the victims but also their families, communities and future generations. Sickness or injury of the main income earner may reduce their ability to provide for the family, including food and education, with inherent negative effects. (*7*) The extra burden placed on family members of caring for the sick or injured person may also negatively affect the family unit. Sick or injured children can lose valuable time away from education, affecting their future, which in turn can negatively affect the family unit and the community. Trained community first responders could reduce this burden.

Additional benefits of community first responders stem from their intimate knowledge of the culture, assets and needs of the community. An example is the work of community rangers in the Treaty Village Resilience Program in Papua New Guinea. (*8*) The rangers work with local nurses to deliver health and nutrition programmes and provide birthing assistance. All rangers complete first aid training and collaborate with villages to deliver projects to improve health, including water and sanitation.

Not only must community first responders understand the culture and needs of communities, but the community must trust the first responder programme and its participants. Trust in community first responders was a key factor in a study in the United Republic of United Republic of Tanzania of the perceptions of trauma patients to the introduction of community first responders. (*9*) Family members and neighbours were trusted most to deliver first aid, and taxi drivers and police officers were considered the least trustworthy. Members of religious groups were also identified as a potential source of first responders, but this recommendation was not tested. Trust in community first responders is poorly understood in the WPR.

Community first responders can also monitor and report important diseases in communities, as evidenced in a recent outbreak of Ebola virus disease in West Africa. Contact tracing and reporting of early symptoms at district and local levels by community and religious leaders helped to identify and contain the outbreak in some communities. (*10*) Community and religious leaders also disseminated information and improved community cooperation in reporting disease presentations. (*10*) This experience shows the importance of local training and capacity-building and of tailoring programmes to the local context. (*11*)

High-income countries in the WPR, such as Australia, have a responsibility to support LMICs in improving their health systems and training community first responders as they move towards achievement of the Sustainable Development Goals. (*12*) The potential of locally designed and developed community first responder programmes to reduce the burden of injury and illness in the WPR is untapped. A first step could be to work with local communities to understand how best to conduct training that is culturally relevant, acceptable and effective. If lives are to be saved and disability reduced, LMICs in the Region must find ways to provide effective training for community first responders, systems to sustain training and monitoring and optimal incorporation of social and cultural contexts into training.
